# Supplementary material for: CUG-binding protein 1 regulates HSC activation and liver fibrogenesis
Source: Nat Commun. 2016 Nov 17;7:13498. doi: 10.1038/ncomms13498 (PMC5118555; doi:10.1038/ncomms13498)
Supplement: Supplementary Information — Supplementary Figures 1-10 and Supplementary Tables 1-3. [file ncomms13498-s1.pdf]

## Supplementary Figures

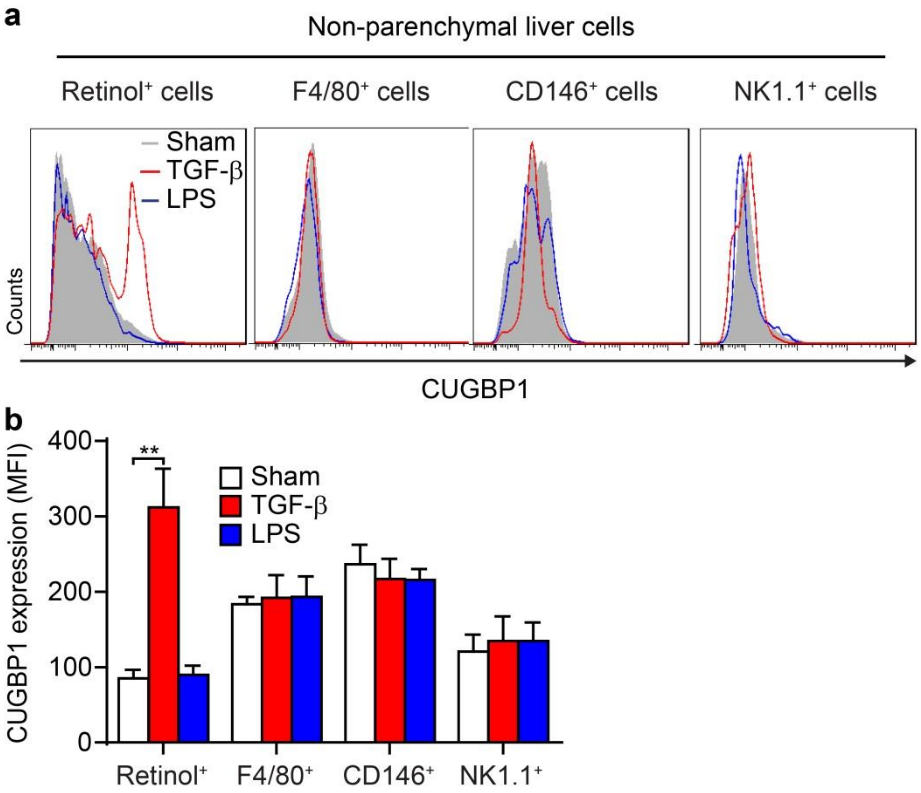


**Supplementary Figure 1. Expression of CUGBP1 in non-parenchymal liver cells treated with TGF-β and LPS.** Non-parenchymal liver cells were isolated and treated with or without TGF-β (10 ng $ml^{-1}$) or LPS (100 ng $ml^{-1}$) (**a** - **b**) Flow cytometry analyses of CUGBP1 expression. (mean ± SEM; n = 5, $^{**}P < 0.01$ as indicated by Student's *t* test). The data are representative of two independent experiments.

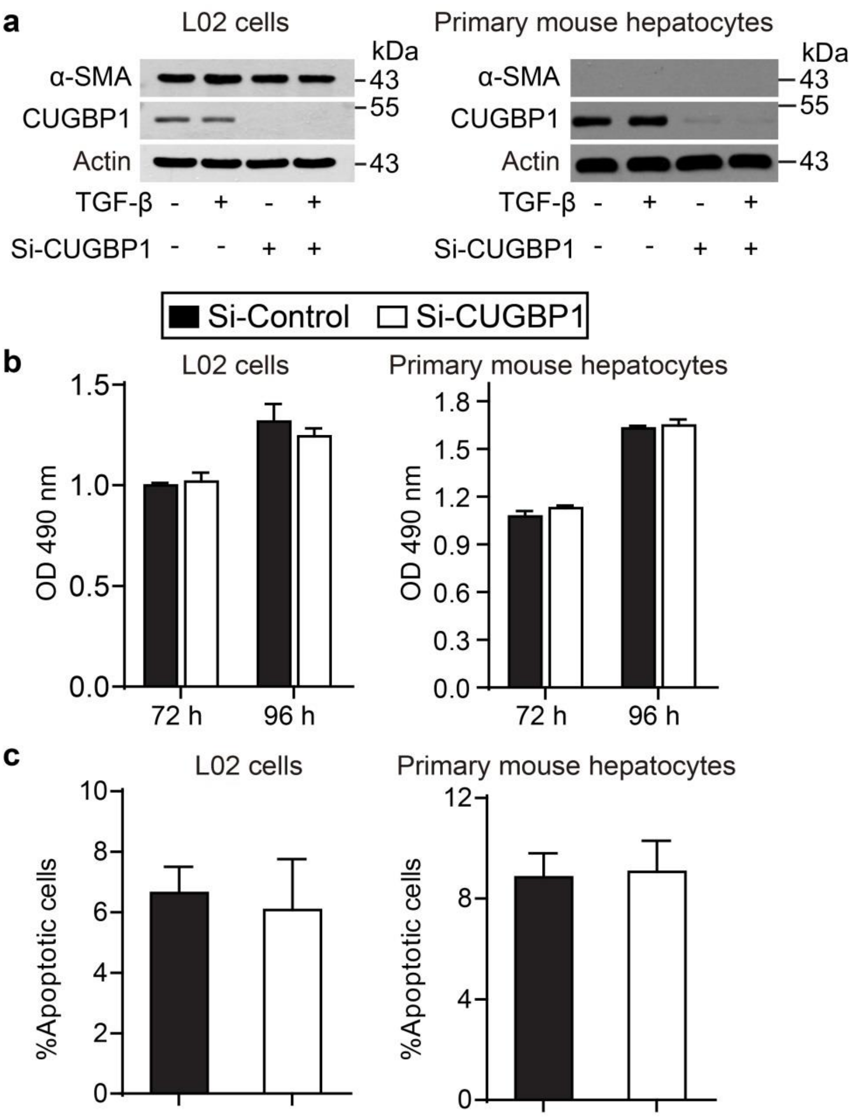


**Supplementary Figure 2. Effect of CUGBP1 on L02 cells and primary mouse hepatocytes.** (**a**) After transfection with si-CUGBP1 or control siRNA for 48 hours, L02 cells and primary mouse hepatocytes were treated with or without 5 ng $ml^{-1}$ TGF-β for 24 hours. Cell extracts were subjected to Western blot analyses. Data are representative of two experiments. (**b**) L02 cells and primary mouse hepatocytes were transfected with si-CUGBP1 or control siRNA for indicated time intervals. The CytoTox 96 assay was carried out to detect the cell number (n = 6). (**c**) Percentages of Annexin V positive cells in L02 cells and primary mouse hepatocytes transfected with si-CUGBP1 or control siRNA for 48 hours (n = 6). (**b** - **c**) Data are given in the form of mean ± SEM of three experiments.

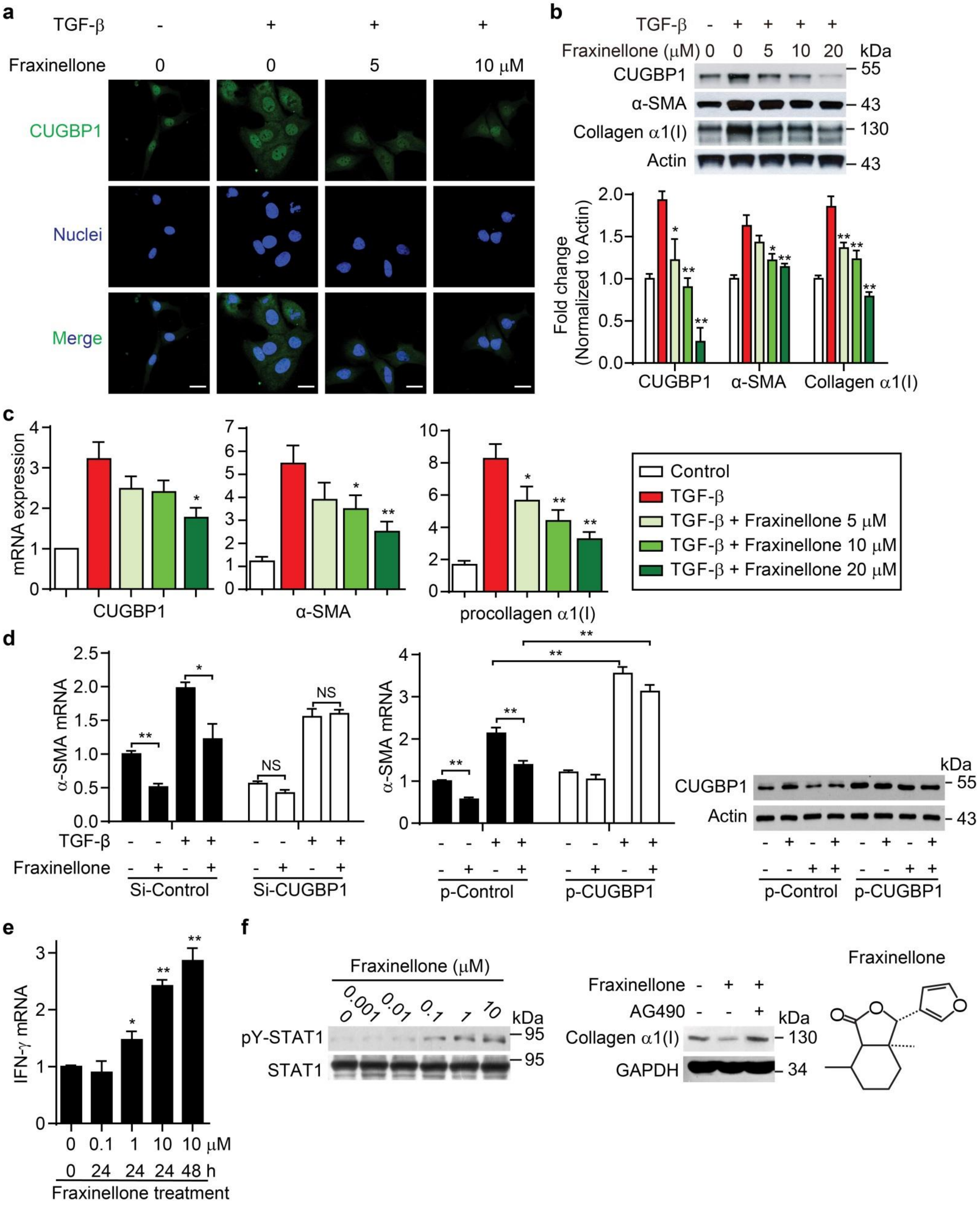


**Supplementary Figure 3. Decrease in CUGBP1 expression in HSCs by fraxinellone inhibits HSC activation via increased IFN-γ production.** LX-2 cells were cultured with fraxinellone (5, or 10 μM) for 6 hours, and treated with TGF-β (5 ng $ml^{-1}$). Cells were harvested for immunofluorescence analyses (Scale

bars, 25 μm). (**a**) and Western blot analyses (**b**) 24 hours later, or for quantitative PCR analyses (**c**) 6 hours later (mean ± SEM; n = 3, **P* < 0.05, ***P* < 0.01 by one-way ANOVA followed by Dunnett's test). (**d**) After transfection with si-CUGBP1 or control siRNA for 48 hours or transfection with control plasmid or pCDNA3.1-CUGBP1 plasmid for 24 hours, LX-2 cells were treated with or without fraxinellone (20 μM) for 6 hours followed by treatment with or without TGF-β (5 ng/ml) for 24 hours. The cells were harvested for quantitative PCR analyses (mean ± SEM; n = 3, **P* < 0.05, ***P* < 0.01 by Student's *t* test; NS, not significant). (**e**) Quantitative PCR analyses of mRNA expression of IFN-γ in LX-2 cells pretreated with fraxinellone at different concentrations for 24 or 48 hours (mean ± SEM; n = 3, **P* < 0.05, ***P* < 0.01 versus medium control by one-way ANOVA followed by Dunnett's test). (**f**) LX-2 cells were treated with various concentrations of fraxinellone for 48 hours, or treated with fraxinellone (10 μM) alone or plus AG490 (5 μM) for up to 48 hours. Expressions of the indicated proteins were analyzed using Western blotting. The chemical structure of fraxinellone was presented in the right side of (**f**). The data are representative of two independent experiments.

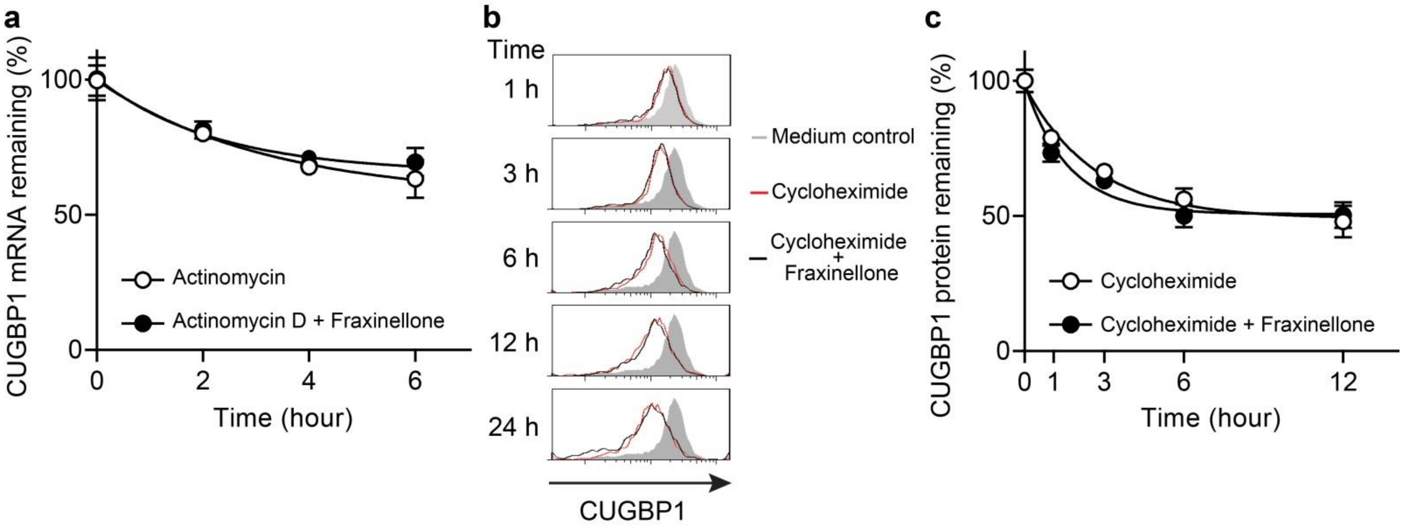


**Supplementary Figure 4. Effects of fraxinellone on the mRNA and protein stability of CUGBP1.** (**a**) LX-2 cells were treated with actinomycin D (1 μg/ml) and with or without fraxinellone (20 μM) for indicated time intervals. And then quantitative PCR was carried out to detect the remaining mRNA expression of CUGBP1 (n = 6). (**b**-**c**) LX-2 cells were treated with cycloheximide (10 μg $ml^{-1}$) and with or without fraxinellone (20 μM) for indicated time intervals. And then cells were stained with anti-CUGBP1 antibody conjugated with Alexa Fluor® 488. (**b**) FACS analysis of CUGPB1 expression. (**c**) Percentages of remaining protein expression of CUGBP1 from (**b**) (n = 6). Data are given in the form of mean ± SEM of three experiments.

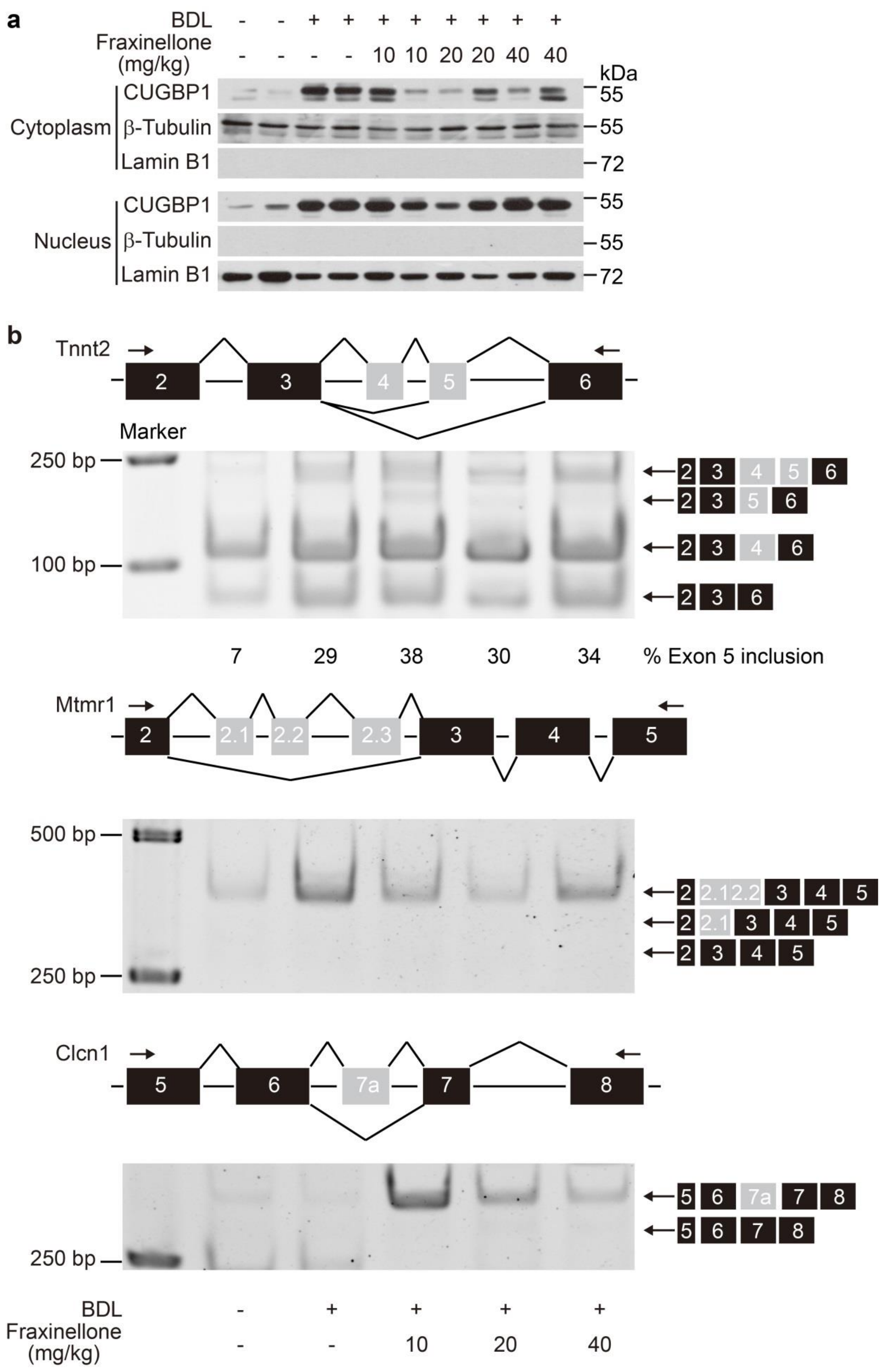


**Supplementary Figure 5. Splicing pattern of CUGBP1 related genes in fibrotic liver.** (**a**) Western blot analyses of liver samples of BDL mice treated as in Figure 7. (**b**) RT–PCR analysis of Tnnt2, Mtmr1, and Clcn1 in the liver of BDL mice. The data are representative of two independent experiments.

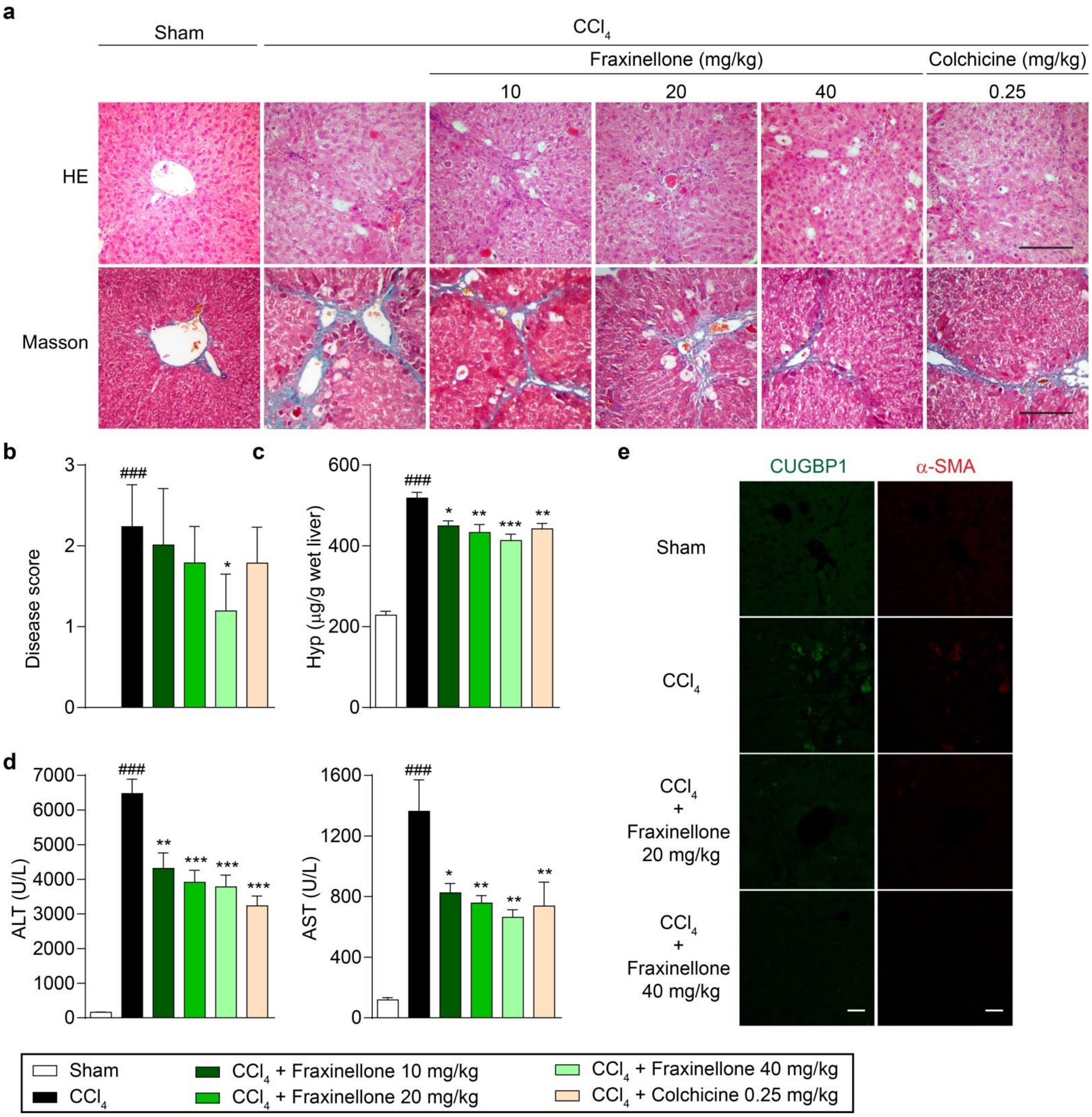


**Supplementary Figure 6. Fraxinellone improved the $CCl_4$-induced liver fibrosis.** Male ICR mice were given daily intragastric administration of 10, 20 and 40 mg kg$^{-1}$ fraxinellone or 0.25 mg kg$^{-1}$ colchicine for 8 weeks. Those in sham and control groups were given equal volume of vehicle solution. After one-week administration, all mice except those in normal group were intraperitoneally injected with $CCl_4$ (0.3% $CCl_4$/olive oil 10 ml kg$^{-1}$ body weight twice a week) for seven weeks. Then, the mice were sacrificed 24 hours after the last injection. The sections of mice liver were stained with (**a**) hematoxylin-eosin or Masson and examined by a blinded histologist (Scale bars, 50 μm). (**b**) Disease score of paraffin wax embedded

liver specimens. (**c**) Expression level of liver Hyp. (**d**) Serum expression levels of ALT and AST. (**e**) IHF analysis of CUGBP1 and α-SMA expression in the paraffin-embedded liver sections (Scale bars, 25 μm). (**b** - **d**, mean ± SEM; n = 8). $^{*}P < 0.05$, $^{**}P < 0.01$, and $^{***}P < 0.001$ versus $CCl_4$ model by one-way ANOVA followed by Dunnett's test, $^{###}P < 0.001$ versus sham by Student's *t* test.

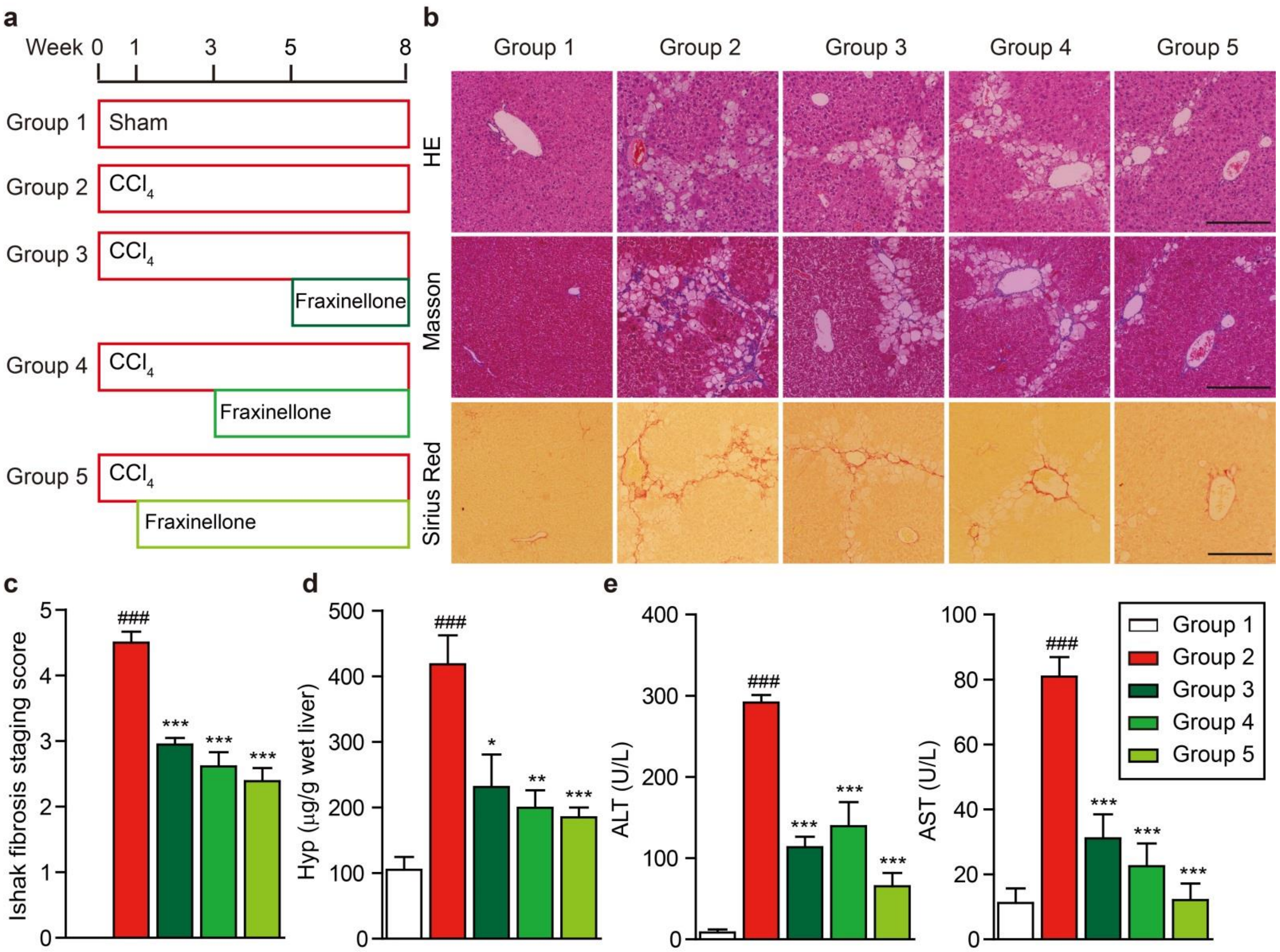


**Supplementary Figure 7. The therapeutic effect of fraxinellone on $CCl_4$-induced liver fibrosis.** Male ICR mice were given daily intragastric administration of 40 mg $kg^{-1}$ fraxinellone for indicated weeks. Those in sham and control groups were given equal volume of vehicle solution. All mice except those in normal group were intraperitoneally injected with $CCl_4$ (0.3% $CCl_4$/olive oil 10 ml $kg^{-1}$ body weight twice a week) for seven weeks. Then, the mice were sacrificed 24 hours after the last injection. The sections of mice liver were stained with (**a**) The timespan of fraxinellone treatments in each group. (**b**) Representative microphotograph of H & E-stained, Masson-stained and Sirius Red-stained paraffin-embedded sections of liver tissues (Scale bars, 100 μm). (**c**) Disease score of paraffin wax embedded liver specimens. (**d**) Expression level of liver Hyp. (**e**) Serum expression levels of ALT and AST. (**c** - **e**, mean ± SEM; n = 8). $^{*}P < 0.05$, $^{**}P < 0.01$, and $^{***}P < 0.001$ versus Group 2 by one-way ANOVA followed by Dunnett's test, $^{###}P < 0.001$ versus sham by Student's *t* test.

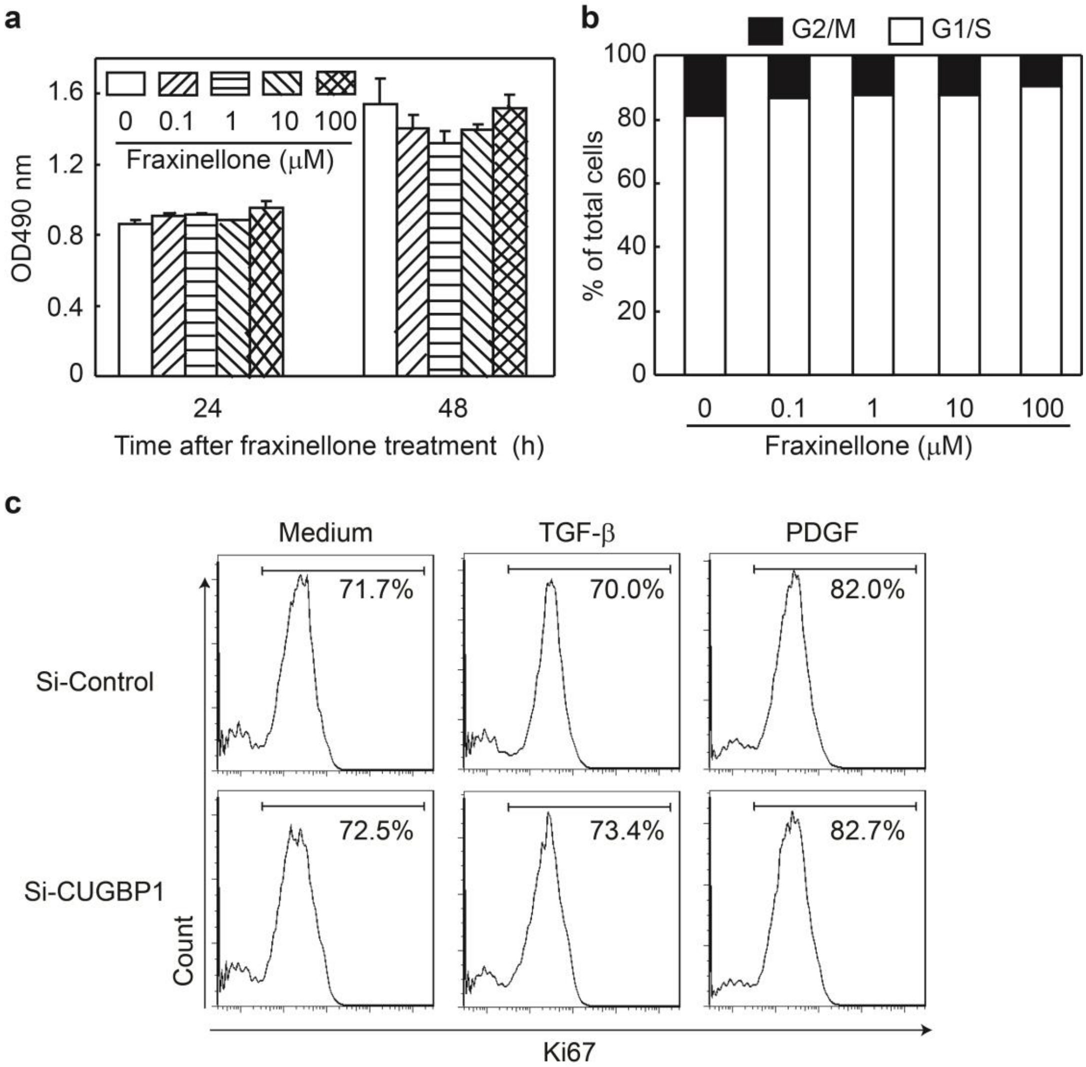


**Supplementary Figure 8. Fraxinellone did not affect the cell viability of LX-2 cells.** (**a**) LX-2 cells were treated with increasing concentrations of fraxinellone for 24 or 48 h. The CytoTox 96 assay was carried out to detect the cell number (n = 9). Data are given in the form of mean ± SEM of three experiments and each experiment includes triplicate wells. (**b**) Cell-cycle distribution of LX-2 cells treated with increasing concentrations of fraxinellone for 48 h. Then, cells were collected, stained with propidium iodide, and subjected to cell-cycle analyses. (**c**) LX-2 cells were transfected with si-CUGBP1 or control siRNA for 48 hours and treated with or without 5 ng $ml^{-1}$ TGF-β or 10 ng $ml^{-1}$ PDGF for 24 hours. Then, cells were subjected to FACS analyses. The data are representative of two independent experiments.

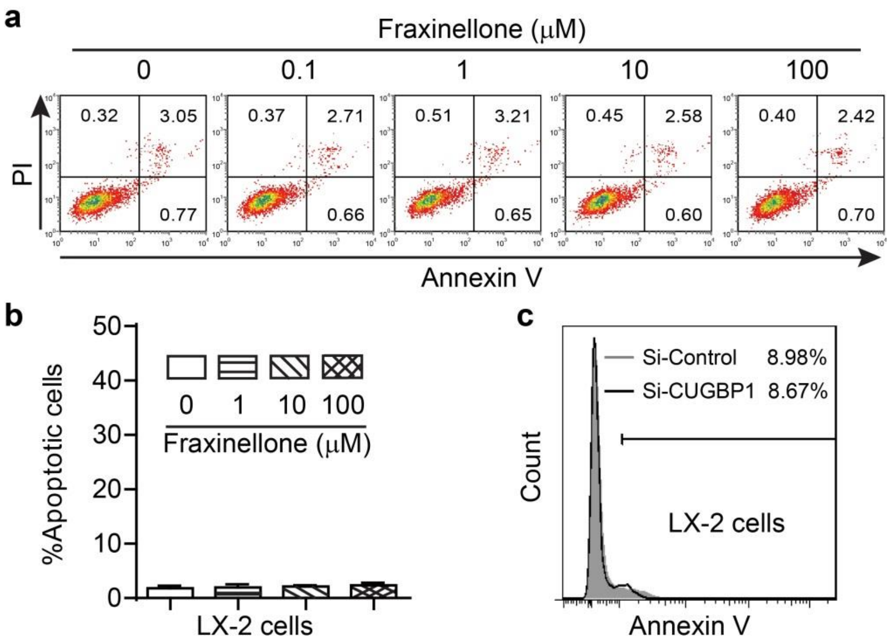


**Supplementary Figure 9. Fraxinellone did not affect the apoptosis of LX-2 cells.** (**a**) LX-2 cells were treated with increasing concentrations of fraxinellone for 24 h. FACS was carried out to detect the percentage of apoptotic cell. (**b**) Graph of the FACS analysis of apoptotic LX-2 cells from (**a**). Data are given in the form of mean ± SEM of three experiments. (**c**) LX-2 cells were transfected with si-CUGBP1 or control siRNA for 48 hours. Then, cells were subjected to FACS analyses of apoptosis. The data are representative of two independent experiments.

**Fig. 2b**

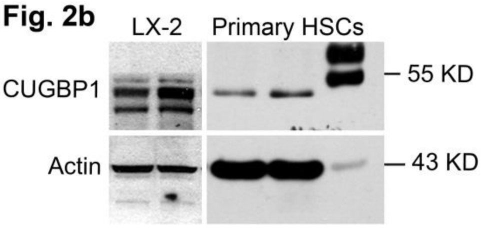


**Fig. 2c**

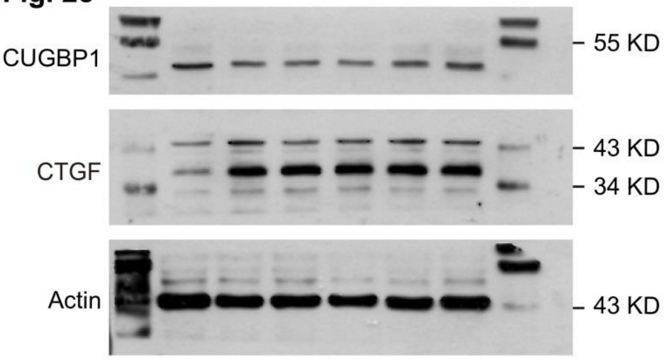


**Fig. 2f**

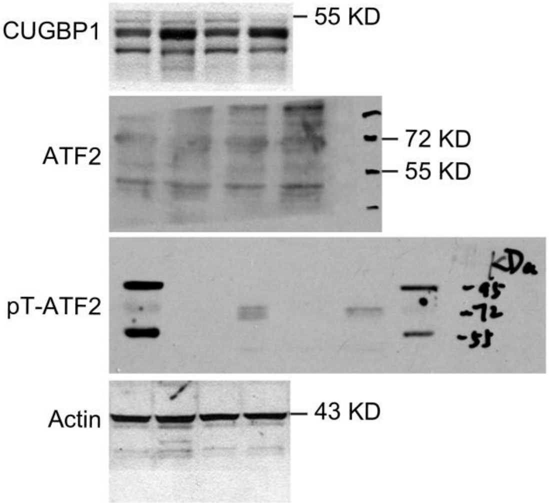


**Fig. 2h**

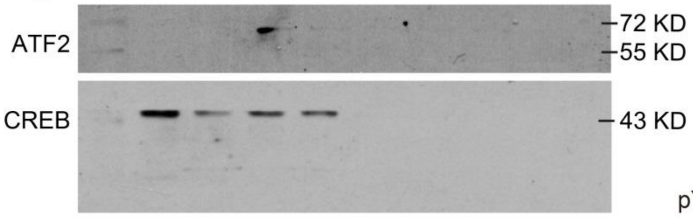


**Fig. 3c**

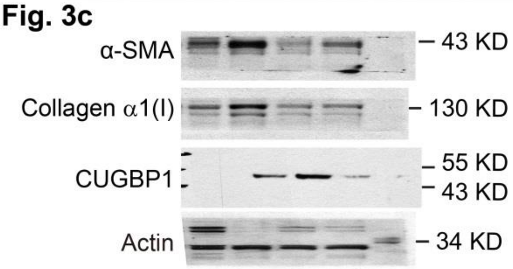


**Fig. 4a**

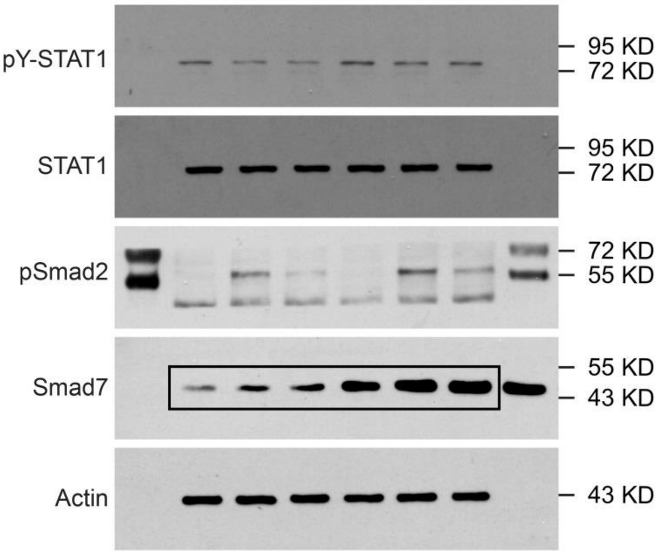


**Fig. 5b**

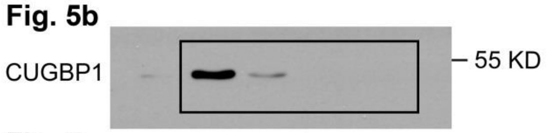


**Fig. 5c**

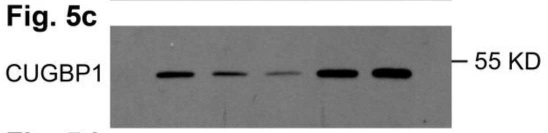


**Fig. 5d**

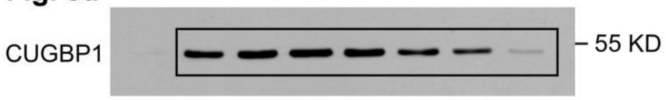


**Fig. 7a**

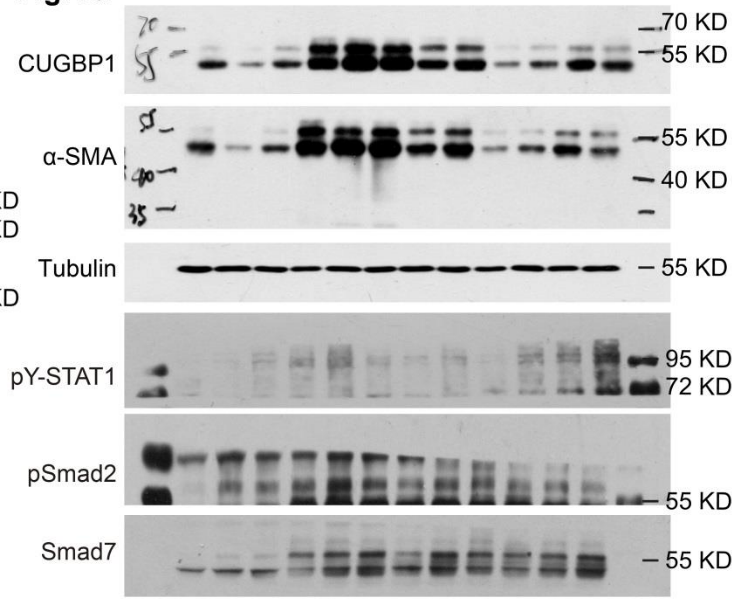

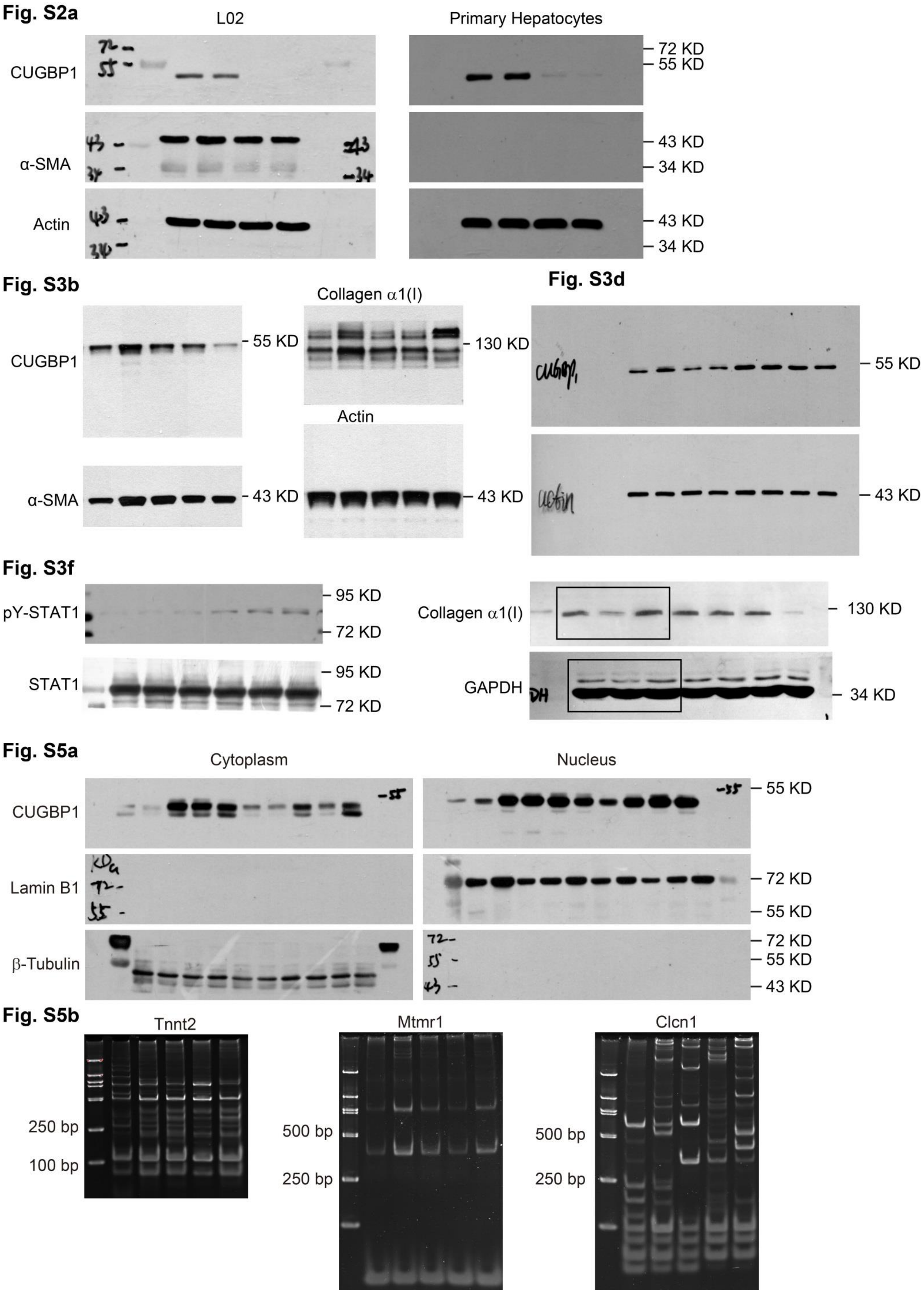


**Supplementary Figure 10.** Uncropped scans of the Western blots shown in the indicated figures.

## Supplementary Tables

| Gene | Gene_id | Readcount_ si-CUGBP1 | Readcount_ si-Control | log2. FC | p value | q value |
|---|---|---|---|---|---|---|
| α-SMA | ENSG00000107796 | 92.206 | 205.229 | -1.154 | 1.69E-07 | 3.10E-06 |
| BMPR2 | ENSG00000204217 | 63.829 | 26.275 | 1.281 | 1.61E-06 | 2.40E-05 |
| Smad5 | ENSG00000113658 | 50.979 | 23.788 | 1.100 | 9.63E-05 | 0.000941 |
| p107 | ENSG00000080839 | 41.074 | 11.624 | 1.821 | 2.08E-06 | 3.02E-05 |
| p300 | ENSG00000005339 | 260.824 | 130.350 | 1.001 | 9.22E-17 | 7.42E-15 |
| ROCK1 | ENSG00000067900 | 86.431 | 15.246 | 2.503 | 4.16E-16 | 3.11E-14 |

**Supplementary Table 1. mRNA expression changes of genes in TGF-β signaling pathway by knockdown of CUGBP1 in activated LX-2 cells.**

| No. | Age | Sex | Organ | Pathology diagnosis | Tissue ID. | Type |
|---|---|---|---|---|---|---|
| 1 | 34 | M | Liver | Cancer adjacent hepatic tissue | Dlv030943 | NAT |
| 2 | 35 | M | Liver | Cancer adjacent hepatic tissue | Dlv030221 | NAT |
| 3 | 64 | M | Liver | Cancer adjacent hepatic tissue | Dlv062184 | NAT |
| 4 | 38 | M | Liver | Cancer adjacent hepatic tissue | Dlv060420 | NAT |
| 5 | 27 | M | Liver | Cancer adjacent hepatic tissue | Dlv031249 | NAT |
| 6 | 52 | F | Liver | Cancer adjacent hepatic tissue | Dlv010201 | NAT |
| 7 | 41 | M | Liver | Cancer adjacent hepatic tissue | Dlv031997 | NAT |
| 8 | 42 | F | Liver | Cancer adjacent hepatic tissue | Dlv031507 | NAT |
| 9 | 38 | F | Liver | Cancer adjacent hepatic tissue | Dlv030490 | NAT |
| 10 | 25 | F | Liver | Cancer adjacent hepatic tissue | Dlv031716 | NAT |
| 11 | 35 | M | Liver | Cancer adjacent hepatic tissue | Dlv031809 | NAT |
| 12 | 56 | M | Liver | Cancer adjacent hepatic tissue | Dlv041091 | NAT |
| 13 | 49 | M | Liver | Cancer adjacent hepatic tissue | Dlv051141 | NAT |
| 14 | 51 | F | Liver | Cancer adjacent hepatic tissue | Dlv061716 | NAT |
| 15 | 65 | F | Liver | Cancer adjacent hepatic tissue | Dlv051119 | NAT |
| 16 | 56 | M | Liver | Cancer adjacent hepatic tissue | Dlv040994 | NAT |
| 17 | 63 | M | Liver | Cancer adjacent hepatic tissue | Dlv031797 | NAT |
| 18 | 35 | F | Liver | Cancer adjacent hepatic tissue | Dlv060974 | NAT |
| 19 | 61 | F | Liver | Cancer adjacent hepatic tissue | Dlv041048 | NAT |
| 20 | 73 | M | Liver | Cancer adjacent hepatic tissue | Dlv041067 | NAT |
| 21 | 32 | M | Liver | Cancer adjacent hepatic tissue | Dlv041167 | NAT |
| 22 | 35 | M | Liver | Cancer adjacent hepatic tissue | Dlv05N006 | normal |
| 23 | 40 | M | Liver | Cancer adjacent hepatic tissue | Dlv05N013 | normal |
| 24 | 38 | M | Liver | Cancer adjacent hepatic tissue | Dlv05N015 | normal |
| 25 | 40 | M | Liver | Cancer adjacent hepatic tissue | Dlv05N014 | normal |
| 26 | 16 | M | Liver | Cancer adjacent hepatic tissue | Dlv06N007 | normal |
| 27 | 45 | M | Liver | Cancer adjacent hepatic tissue | Dlv06N001 | normal |
| 28 | 35 | F | Liver | Cancer adjacent hepatic tissue | Dlv03N009 | normal |
| 29 | 21 | F | Liver | Cancer adjacent hepatic tissue | Dlv05N001 | normal |
| 30 | 50 | F | Liver | Cancer adjacent hepatic tissue | Dlv03N005 | normal |
| 31 | 45 | M | Liver | Chronic hepatitis | Dlv030361 | inflammation |
| 32 | 43 | M | Liver | Chronic hepatitis | Dlv022879 | inflammation |
| 33 | 47 | F | Liver | Chronic hepatitis with fatty degeneration | Dlv024176 | inflammation |
| 34 | 41 | M | Liver | Chronic hepatitis | Dlv030358 | inflammation |
| 35 | 59 | F | Liver | Chronic hepatitis | Dlv031753 | inflammation |
| 36 | 50 | M | Liver | Chronic hepatitis | Dlv031874 | inflammation |
| 37 | 51 | M | Liver | Chronic hepatitis | Dlv021087 | inflammation |
| 38 | 58 | M | Liver | Chronic hepatitis | Dlv051217 | inflammation |
| 39 | 50 | M | Liver | Chronic hepatitis | Dlv041081 | inflammation |
| 40 | 65 | M | Liver | Chronic hepatitis | Dlv040078 | inflammation |
| 41 | 49 | M | Liver | Hepatic cirrhosis | Dlv031740 | Cirrhosis |
| 42 | 42 | M | Liver | Hepatic cirrhosis | Dlv010124 | Cirrhosis |
| 43 | 43 | M | Liver | Hepatic cirrhosis | Dlv010288 | Cirrhosis |
| 44 | 48 | M | Liver | Hepatic cirrhosis | Dlv031031 | Cirrhosis |
| 45 | 67 | M | Liver | Hepatic cirrhosis | Dlv010874 | Cirrhosis |

| | | | | | | |
|---|---|---|---|---|---|---|
| 46 | 57 | M | Liver | Hepatic cirrhosis | Dlv011339 | Cirrhosis |
| 47 | 33 | M | Liver | Hepatic cirrhosis | Dlv031799 | Cirrhosis |
| 48 | 55 | M | Liver | Hepatic cirrhosis | Dlv022056 | Cirrhosis |
| 49 | 39 | M | Liver | Hepatic cirrhosis | Dlv041496 | Cirrhosis |
| 50 | 38 | M | Liver | Hepatic cirrhosis | Dlv022086 | Cirrhosis |
| 51 | 51 | M | Liver | Hepatic cirrhosis | Dlv022101 | Cirrhosis |
| 52 | 34 | M | Liver | Hepatic cirrhosis | Dlv041727 | Cirrhosis |
| 53 | 48 | M | Liver | Hepatic cirrhosis | Dlv022148 | Cirrhosis |
| 54 | 48 | M | Liver | Hepatic cirrhosis | Dlv022151 | Cirrhosis |
| 55 | 40 | M | Liver | Hepatic cirrhosis | Dlv022889 | Cirrhosis |
| 56 | 40 | M | Liver | Hepatic cirrhosis | Dlv022892 | Cirrhosis |
| 57 | 46 | M | Liver | Hepatic cirrhosis | Dlv023412 | Cirrhosis |
| 58 | 39 | M | Liver | Hepatic cirrhosis | Dlv023420 | Cirrhosis |
| 59 | 51 | M | Liver | Hepatic cirrhosis | Dlv023640 | Cirrhosis |
| 60 | 51 | M | Liver | Hepatic cirrhosis | Dlv023644 | Cirrhosis |
| 61 | 35 | M | Liver | Hepatic cirrhosis | Dlv023651 | Cirrhosis |
| 62 | 39 | F | Liver | Hepatic cirrhosis | Dlv023921 | Cirrhosis |
| 63 | 48 | M | Liver | Hepatic cirrhosis | Dlv023927 | Cirrhosis |
| 64 | 45 | F | Liver | Hepatic cirrhosis | Dlv030258 | Cirrhosis |
| 65 | 31 | M | Liver | Hepatic cirrhosis | Dlv031817 | Cirrhosis |
| 66 | 40 | M | Liver | Hepatic cirrhosis | Dlv024196 | Cirrhosis |
| 67 | 60 | M | Liver | Hepatic cirrhosis | Dlv024197 | Cirrhosis |
| 68 | 68 | F | Liver | Hepatic cirrhosis | Dlv024306 | Cirrhosis |
| 69 | 51 | M | Liver | Hepatic cirrhosis | Dlv024312 | Cirrhosis |
| 70 | 47 | M | Liver | Hepatic cirrhosis | Dlv024484 | Cirrhosis |
| 71 | 57 | M | Liver | Hepatic cirrhosis | Dlv062670 | Cirrhosis |
| 72 | 49 | M | Liver | Hepatic cirrhosis | Dlv030086 | Cirrhosis |
| 73 | 57 | M | Liver | Hepatic cirrhosis | Dlv050119 | Cirrhosis |
| 74 | 47 | M | Liver | Hepatic cirrhosis | Dlv030194 | Cirrhosis |
| 75 | 23 | M | Liver | Hepatic cirrhosis | Dlv030200 | Cirrhosis |
| 76 | 62 | F | Liver | Hepatic cirrhosis | Dlv030209 | Cirrhosis |
| 77 | 58 | M | Liver | Hepatic cirrhosis | Dlv051446 | Cirrhosis |
| 78 | 60 | M | Liver | Hepatic cirrhosis | Dlv062596 | Cirrhosis |
| 79 | 38 | M | Liver | Hepatic cirrhosis | Dlv030270 | Cirrhosis |
| 80 | 53 | M | Liver | Hepatic cirrhosis | Dlv060134 | Cirrhosis |

**Supplementary Table 2. Tissue array patient information.**

| Gene | Forward primer sequence (5'-3') | Reverse primer sequence (5'-3') |
|---|---|---|
| Human CUGBP1 | ACATCCGAGTCATGTTCTCTTCG | CATTGCCTTGATAGCCGTCTG |
| Human α-SMA | CTATGAGGGCTATGCCTTGCC | GCTCAGCAGTAGTAACGAAGGA |
| Human IFN-γ | TCGGTAACTGACTTGAATGTCCA | TCGCTTCCCTGTTTTAGCTGC |
| Human Smad7 | TTCCTCCGCTGAAACAGGG | CCTCCCAGTATGCCACCAC |
| Human COL1A1 | ATCAACCGGAGGAATTTCCGT | CACCAGGACGACCAGGTTTTC |
| Mouse α-SMA | GTCCCAGACATCAGGGAGTAA | TCGGATACTTCAGCGTCAGGA |
| Mouse COL3A1 | CTGTAACATGGAAACTGGGGAAA | CCATAGCTGAACTGAAAACCACC |
| Mouse COL4A1 | CTGGCACAAAAGGGACGAG | ACGTGGCCGAGAATTTCACC |
| Human GAPDH | AACGACCCCTTCATTGAC | TCCACGACATACTCAGCAC |
| Mouse GAPDH | AGGTCGGTGTGAACGGATTTG | TGTAGACCATGTAGTTGAGGTCA |

**Supplementary Table 3. Primer sequences for PCR.**
